# Effect of low‐to‐moderate‐dose corticosteroids on mortality of hospitalized adolescents and adults with influenza A(H1N1)pdm09 viral pneumonia

**DOI:** 10.1111/irv.12456

**Published:** 2017-06-09

**Authors:** Hui Li, Shi‐gui Yang, Li Gu, Yao Zhang, Xi‐xin Yan, Zong‐an Liang, Wei Zhang, Hong‐yu Jia, Wei Chen, Meng Liu, Kai‐jiang Yu, Chun‐xue Xue, Ke Hu, Qi Zou, Lan‐juan Li, Bin Cao, Chen Wang, the National Influenza A(H1N1)pdm09 Clinical Investigation Group of China

**Affiliations:** ^1^ Beijing Chao‐Yang HospitalCapital Medical UniversityBeijingChina; ^2^ State Key Laboratory for Diagnosis and Treatment of Infectious DiseasesCollaborative Innovation Center for Diagnosis and Treatment of Infectious DiseasesThe First Affiliated HospitalCollege of MedicineZhejiang UniversityHangzhouChina; ^3^ Global Health, Population & NutritionGlobal Research & ServicesFamily Health International 360DurhamNCUSA; ^4^ Department of Respiratory MedicineSecond Hospital of Hebei Medical UniversityShijiazhuangChina; ^5^ West China Hospital of Sichuan UniversityChengduChina; ^6^ The First Affiliated HospitalNanchang UniversityNanchangChina; ^7^ Shengjing Hospital of China Medical UniversityShenyangChina; ^8^ The Second Affiliated Hospital of Harbin Medical UniversityHarbinChina; ^9^ Renmin Hospital of Wuhan UniversityWuhanChina; ^10^ Fujian Medical University Union HospitalFuzhouChina; ^11^ Center for Respiratory Diseases China‐Japan Friendship HospitalBeijingChina; ^12^ Department of Respiratory MedicineCapital Medical UniversityBeijingChina; ^13^ National Clinical Research Centre for Respiratory DiseaseBeijingChina; ^14^ Department of Pulmonary and Critical Care MedicineChina‐Japan Friendship HospitalBeijingChina

**Keywords:** corticosteroids, influenza A(H1N1)pdm09 virus, mortality, pneumonia

## Abstract

**Background:**

The effect of corticosteroids on influenza A(H1N1)pdm09 viral pneumonia patients remains controversial, and the impact of dosage has never been studied.

**Methods:**

Using data of hospitalized adolescent and adult patients with influenza A(H1N1)pdm09 viral pneumonia, prospectively collected from 407 hospitals in mainland China, the effects of low‐to‐moderate‐dose (25‐150 mg d^−1^) and high‐dose (>150 mg d^−1^) corticosteroids on 30‐day mortality, 60‐day mortality, and nosocomial infection were assessed with multivariate Cox regression and propensity score‐matched case–control analysis.

**Results:**

In total, 2141 patients (median age: 34 years; morality rate: 15.9%) were included. Among them, 1160 (54.2%) had PaO_2_/FiO_2_<300 mm Hg on admission, and 1055 (49.3%) received corticosteroids therapy. Corticosteroids, without consideration of dose, did not influence either 30‐day or 60‐day mortality. Further analysis revealed that, as compared with the no‐corticosteroid group, low‐to‐moderate‐dose corticosteroids were related to reduced 30‐day mortality (adjusted hazard ratio [aHR] 0.64 [95% CI 0.43‐0.96, *P*=.033]). In the subgroup analysis among patients with PaO_2_/FiO_2_<300 mm Hg, low‐to‐moderate‐dose corticosteroid treatment significantly reduced both 30‐day mortality (aHR 0.49 [95% CI 0.32‐0.77]) and 60‐day mortality (aHR 0.51 [95% CI 0.33‐0.78]), while high‐dose corticosteroid therapy yielded no difference. For patients with PaO_2_/FiO_2_ ≥300 mm Hg, corticosteroids (irrespective of dose) showed no benefit and even increased 60‐day mortality (aHR 3.02 [95% CI 1.06‐8.58]). Results were similar in the propensity model analysis.

**Conclusions:**

Low‐to‐moderate‐dose corticosteroids might reduce mortality of influenza A(H1N1)pdm09 viral pneumonia patients with PaO_2_/FiO_2_<300 mm Hg. Mild patients with PaO_2_/FiO_2_ ≥300 mm Hg could not benefit from corticosteroid therapy.

## 1 INTRODUCTION

Corticosteroids were widely used in patients with severe influenza infection. The use of corticosteroids is based on the underlying pathogenesis of severe influenza infection, which is characterized by overproduction of proinflammatory cytokines/chemokines^1^, ^2^ and an excess of activated lymphocytes,^3^ which may result in severe lung damage^4^, ^5^ and delayed recovery.^6^ Moreover, animal model studies also found that corticosteroid treatment decreased mortality and ameliorated the acute lung injury induced by influenza A(H1N1)pdm09 virus.^7^, ^8^ Although corticosteroids are posited to be beneficial in the treatment of severe influenza patients, clinical results remain contradictory and opposition overwhelms support in studies published so far.^9^, ^10^, ^11^, ^12^, ^13^, ^14^, ^15^, ^16^, ^17^ It is reported that corticosteroid might impair viral clearance and increase secondary infection.^13^, ^18^, ^19^ The major guidelines recommend avoiding the use of systemic corticosteroids beyond special occasions such as adrenal insufficiency and severe COPD.^20^

This discrepancy between the “reality” and theory might be translated to the following conditions. First, corticosteroid is definitely not effective at all for all influenza patients due to its side effects. Second, the truth is covered by some confounders, due to that corticosteroid tends to be given to more severe patients. Third, corticosteroid might be only effective at a certain dosage for a specific group of influenza patients who are characterized with severe cytokine storm. However, it is not useful or even harmful for patients otherwise. Therefore, the additive effect on mortality of all patients might be neutral or even harmful, as previously reported.^17^, ^21^

Although the benefits of corticosteroids therapy were recently reported in randomized controlled trials (RCT) and observational studies in patients with severe community‐acquired pneumonia,^22^, ^23^, ^24^ no RCT about the effect of corticosteroids on mortality of influenza‐infected patients has been completed. The majority of researches published so far are retrospective observational studies,^12^, ^13^, ^14^, ^25^, ^26^, ^27^, ^28^, ^29^, ^30^ and most of them involved a relatively small sample. Although meta‐analysis could counteract the small sample size, lack of consistency between studies would preclude any firm conclusions.^17^, ^21^ Moreover, drug dosage was not clearly demonstrated.^17^, ^21^ Studies on acute lung injury (ALI) or acute respiratory distress syndrome (ARDS) revealed that low‐to‐moderate dose corticosteroids reduced mortality significantly,^31^, ^32^ but high‐dose (>2.0 mg·kg^−1^·d^−1^ methylprednisolone) corticosteroid therapy had no such benefits.^31^ However, the effect of low‐to‐moderate‐dose corticosteroids on patients with influenza A(H1N1)pdm09 viral pneumonia has not been determined.

Using the largest nationwide database,^33^, ^34^ comprising of 2141 hospitalized adolescent and adult patients with influenza A(H1N1)pdm09 viral pneumonia, we explored the effect of different doses of adjuvant corticosteroid therapy on mortality of these patients with different disease severity.

## 2 METHODS

### 2.1 Study population and data collection

This study was a retrospective database analysis approved by the research ethics committee of Beijing Chao‐Yang Hospital (10‐ke‐17). Requirement for written informed patient content was waived, because all data were anonymous.

Data was collected prospectively from September 1, 2009 to December 31, 2009, under the surveillance of the Ministry of Health, which has been clearly described in our previous studies.^34^ For each patient, influenza A(H1N1)pdm09 viral infection was confirmed by polymerase chain reaction (PCR) from nasopharyngeal swab, sputum, or bronchoalveolar lavage fluid. To ensure accuracy, data were re‐checked before analysis by two independent researchers.

### 2.2 Endpoints and definitions

Patients aged >14 years with influenza A(H1N1)pdm09 viral pneumonia and complete clinical information were included in the analysis. The primary outcome under investigation was all‐cause mortality within 30 days of disease onset (30‐day mortality). The secondary outcomes included mortality within 60 days (60‐day mortality) and nosocomial infection.

Pneumonia was defined as an acute lower respiratory illness with an opacity or infiltrate seen on chest radiography, which was interpreted as pneumonia by the treating physician.^29^ Nosocomial infection was diagnosed when a positive culture of a new pathogen was obtained from a lower respiratory tract specimen (sputum, bronchial/tracheal aspirates, or bronchoalveolar lavage fluid) and/or blood samples taken ≥48 hours after admission.^13^, ^35^ Invasive pulmonary aspergillosis and mucormycosis were diagnosed in accordance with the revised definitions of invasive fungal diseases from the European Organization for Research and Treatment of Cancer/Invasive Fungal Infections Cooperative Group and the National Institute of Allergy and Infectious Diseases Mycoses Study Group (EORTC/MSG) Consensus Group.^36^ Systemic corticosteroid treatment was defined as any intended therapeutic use of corticosteroids, including methylprednisolone, prednisolone, dexamethasone, and/or hydrocortisone via oral or intravenous routes during the disease course, but excluding inhalational therapy. Low‐to‐moderate‐dose corticosteroids were defined as 25‐150 mg d^−1^ methylprednisolone or equivalent, while high‐dose corticosteroids were defined as >150 mg d^−1^ methylprednisolone or equivalent.^24^, ^31^, ^32^ Patients without corticosteroid treatment were classified as the control group. Neuraminidase inhibitor (NAI) treatment referred to administration of any dose of oseltamivir, zanamivir, and/or peramivir during hospitalization.

### 2.3 Study design and statistical analysis

Continuous variables were summarized as medians (interquartile ranges). For categorical variables, the percentage of patients in each category was calculated. Clinical characteristics were compared between patients who received systemic corticosteroid (corticosteroids) and those not (no corticosteroids), using the Mann‐Whitney *U* test and chi‐squared test as appropriate.

Independent factors were determined by multivariate Cox regression models, including all covariates with *P*<.20 selected from a predetermined list (Table 1) by means of forward logistic regression. The probability for the entry and stepwise removal was 0.05 and 0.10, respectively. All these independent factors were further used to adjust the effects of systemic corticosteroids. Subgroup analysis was performed for patients with influenza A(H1N1)pdm09 viral pneumonia with PaO_2_/FiO_2_<300 mm Hg and PaO_2_/FiO_2_ ≥300 mm Hg on admission.

**Table 1:** Characteristics of 2141 patients hospitalized with influenza A(H1N1)pdm09 viral pneumonia stratified according to the patients’ corticosteroids status

	All patients (n=2141)	Corticosteroids (n=1055)	No corticosteroids (n=1086)	*P*‐value^a^
Hospital category				
Secondary hospital	254 (11.9)	117 (11.1)	137 (12.6)	.275
Tertiary hospital	1887 (88.1)	938 (88.9)	949 (87.4)	
Age, year	34.4 (24.1‐51.1)	35.0 (23.8‐52.4)	33.7 (24.6‐48.7)	.199
14‐20	271 (12.7)	162 (14.9)	109 (10.3)	<.001
21‐40	1049 (49)	481 (44.3)	568 (53.8)	
41‐60	579 (27)	306 (28.2)	273 (25.9)	
61‐80	218 (10.2)	124 (11.4)	94 (8.9)	
>80	24 (1.1)	13 (1.2)	11 (1.0)	
Female	1046 (48.9)	525 (49.8)	521 (48.0)	.408
Underlying diseases				
Hypertension	326 (15.2)	158 (15.0)	168 (15.5)	.751
Diabetes	168 (7.8)	78 (7.4)	90 (8.3)	.442
Cardiovascular disease^b^	131 (6.1)	56 (5.3)	75 (6.9)	.123
COPD	106 (5)	59 (5.6)	47 (4.3)	.177
Asthma	38 (1.8)	22 (2.1)	16 (1.5)	.284
Chronic renal disease	74 (3.5)	36 (3.4)	38 (3.5)	.913
Malignancy^c^	54 (2.5)	28 (2.7)	26 (2.4)	.701
I mmunosuppressive conditions^d^	49 (2.3)	34 (3.2)	15 (1.4)	.004
Pregnancy or post‐partum	452 (21.1)	270 (25.6)	182 (16.8)	<.001
Main laboratory findings on admission				
Leukocytosis	345/2092 (16.5)	191/1037 (18.4)	154/1055 (14.6)	.019
Leukocytopenia	527/2092 (25.2)	267/1037 (25.7)	260/1055 (24.6)	.578
Lymphocytopenia	852/2009 (42.4)	541/994 (54.4)	311/1015 (30.6)	<.001
Hemoglobin, g/L	128 (108‐144)	127 (105‐145)	129 (109‐143)	.091
Platelets, 10^9^/L	152 (113‐200)	141 (104‐187)	163 (125‐209)	<.001
Creatinine, μmol/L	70 (54.5‐88)	69 (54‐90)	70.9 (55.3‐86.8)	.951
PaO_2_/FiO_2_, mm Hg	219.5 (127.9‐326.4)	173.3 (100‐272.4)	286.2 (191.7‐388.2)	<.001
Alanine aminotransferase, U/L	29 (17‐51)	35 (21‐62.3)	24 (15‐43)	<.001
Lactate dehydrogenase, U/L	316 (213‐542)	439 (269‐724)	257 (184‐373)	<.001
Shock	131/2132 (6.1)	84/1052 (8.0)	47/1080 (4.4)	<.001
Invasive mechanical ventilation	416 (19.4)	367 (34.8)	49 (4.5)	<.001
NAI treatment	2047 (95.6)	1025 (97.2)	1022 (94.1)	.001
Interval between symptom onset and NAI treatment, days	6 (4‐7)	6 (4‐8)	5 (3‐7)	<.001
Interval between symptom onset and hospitalization, days	5 (3‐7)	5 (4‐7)	5 (3‐7)	<.001
Antibiotics	2092 (97.7)	1037 (98.3)	1055 (97.1)	.076
ICU admission	960 (44.8)	678 (64.3)	282 (26.0)	<.001
30‐day mortality	306 (14.3)	232 (22.0)	74 (6.8)	<.001
60‐day mortality	337 (15.7)	261(24.7)	76 (7.0)	<.001

COPD, chronic obstructive pulmonary disease; NAI, Neuraminidase inhibitors.

Continuous variables are summarized as median (interquartile range), and categorical variables are presented as number (percentage).

a Mann‐Whitney U‐tests for continuous variables and the chi‐squared tests for categorical variables.

b Cardiovascular disease: including congestive heart disease, coronary atherosclerotic heart disease, and valvular heart disease.

c Malignancy: cancer or hematologic malignancy.

d Immunosuppressive condition: chemotherapy or radiotherapy within 1 mo before the onset of illness, or glucocorticoid therapy (equivalent of 30 mg of prednisone per day for 15 continuous days before the onset of illness).

To minimize the effect of selection bias, we further performed one‐to‐one matching, based on propensity score analysis between patients with low‐to‐moderate‐dose corticosteroids and control, and between patients with high‐dose corticosteroids and control. The propensity scores were determined using multiple logistic regression without regard to outcome. All pre‐specified co‐variables related to the propensity for mortality or for receiving corticosteroids were included in the final models. C‐statistics was used to assess model discrimination. To develop propensity score‐matched pairs without replacement (a 1:1 match), the greedy 5 to 1 digit match algorithm was used, as previously described.^13^ We assessed the balance of baseline covariates between the two treatment groups using the Wilcoxon signed‐rank test for continuous variables and McNemar's test for binary categorical variables. In the propensity‐matched cohort, the Kaplan‐Meier method with log‐rank testing was used to assess differences in mortality between corticosteroid groups and controls.

All tests were two‐tailed, with the significance level set at 0.05. All analyses were carried out using SPSS software for Windows (release 17.0) and SAS version 9.1 (SAS Institute, Cary, NC).

## 3 RESULTS

### 3.1 Clinical characteristics of the cohort

**Figure 1:**
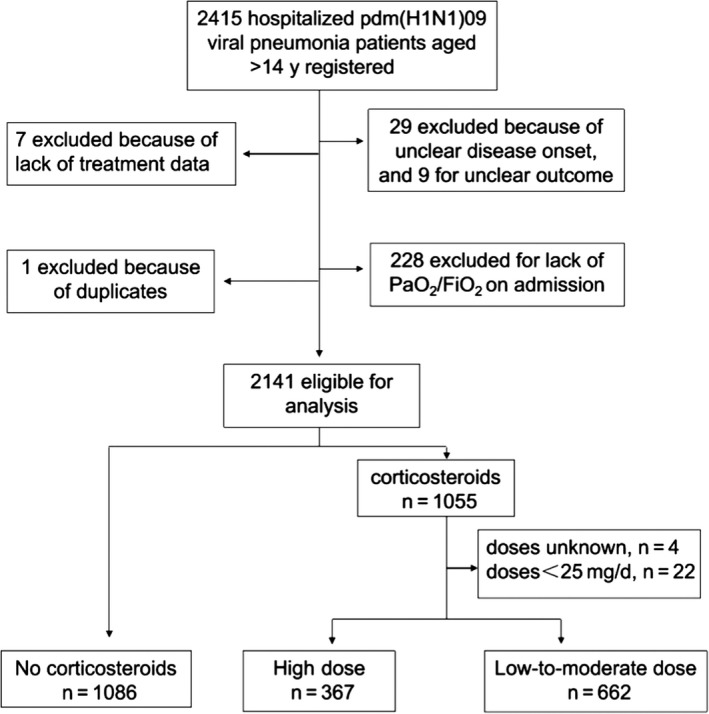
Study flow diagram

During the 4‐month study period, 2415 hospitalized patients with influenza A(H1N1)pdm09 viral pneumonia were registered, of which 274 were excluded for lack of important clinical information or because of duplicate entries (Figure 1). Altogether, 2141 hospitalized cases from 407 hospitals in 27 provinces or municipalities in mainland China were enrolled for final analysis (Table S1).

The median age of the cohort was 34.4 years, and 89% of all the patients were younger than 60 years. The patients were hospitalized within a median duration of 5 days after disease onset, and PaO_2_/FiO_2_ on admission was<300 mm Hg in 54.2% of these patients. The mortality rates of patients with PaO_2_/FiO_2_<100 mm Hg, 100‐200 mm Hg, 200‐300 mm Hg, and ≥300 mm Hg were 48.4%, 23.8%, 16.9%, and 3.5%, respectively. At least one type of antibiotic was administered to 97.7% patients on admission. Oseltamivir was the only NAI used and was given to 95.6% patients within a median duration of 6 days after disease onset (Table 1).

### 3.2 Corticosteroid treatment

Among the 2141 enrolled patients, 1055 (49.3%) received corticosteroid treatment, among whom 939 (89%) patients were administered methylprednisolone, 85 (8.1%) dexamethasone, and 21(2.0%) hydrocortisone. Of them, 662 patients received low‐to‐moderate‐dose corticosteroids and 367 cases received high‐dose corticosteroids (Figure 1). Corticosteroids were initiated within 6 (IQR 4‐8) days after the onset of illness, that is, within 48 hours of admission; baseline values of time‐dependent variables could thus be substituted with values on admission. Patients were treated with corticosteroids for a median duration of 7 (IQR 4‐8) days (Table 2).

**Table 2:** Details of corticosteroid administration in 1055 hospitalized patients with influenza A(H1N1)pdm09 viral pneumonia

Variables	Values
Drug administered, n (%)	
Methylprednisolone	939 (89.0)
Dexamethasone	85 (8.1)
Hydrocortisone	21 (2.0)
Prednisolone	10 (0.9)
Daily dose (equivalent methylprednisolone), mg d^−1^	
Mean±SD	141.3±142
Median (IQR)	80 (53.3‐160)
Time to initiation from the onset of illness, days	
Mean±SD	6.7±4
Median (IQR)	6 (4‐8)
Duration of therapy, days	
Mean±SD	7.7±6.8
Median (IQR)	7 (4‐8)

SD, standard deviation; IQR, interquartile range.

### 3.3 Effect of corticosteroids on mortality by Cox regression analysis

Univariate analysis (Table S2) showed that many factors other than corticosteroid treatment were correlated with mortality, such as underlying comorbidities, baseline disease severity, NAI treatment, and time duration from disease onset to hospitalization. Covariates (Table 1) with *P*<.20 were included in the Cox regression model to determine independent risk factors, all of which were further used to adjust the effect of systemic corticosteroids. Corticosteroids, without consideration of dose, did not influence either the 30‐day or 60‐day mortality. Further analysis showed that, as compared with the control group, low‐to‐moderate‐dose corticosteroids could reduce the 30‐day mortality of patients with influenza A(H1N1)pdm09 viral pneumonia (adjusted hazard ratio [aHR] 0.64 [95% CI 0.43‐0.96, *P*=.033]), while the 60‐day mortality was similar between the two dosage groups and control (Table 3).

**Table 3:** Effect of corticosteroids on mortality of patients hospitalized with influenza A(H1N1)pdm09 viral pneumonia by multivariate Cox regression analysis

	30‐Day Mortality				60‐Day Mortality			
	No. of Patients^a^		Adjusted HR (95% CI)	*P*‐value	No. of Patients^a^		Adjusted HR (95% CI)	*P*‐value
	Corticosteroids	Control			Corticosteroids	Control		
All patients								
Corticosteroids vs control	232/1055	74/1086	0.80 (0.56‐1.15)	.230	261/1055	76/1086	0.87 (0·61‐1.24)	.433
Low‐to‐moderate dose vs control	113/662	74/1086	0.64 (0.43‐0.96)	.033	130/662	76/1086	0.70 (0·47‐1.04)	.078
High‐dose vs control	114/367	74/1086	0.91 (0.58‐1.44)	.694	126/367	76/1086	0.97 (0.62‐1.51)	.882
PaO_2_/FiO_2_ ≥300 mm Hg								
Corticosteroids vs control	17/295	12/686	2.43 (0.82‐7.15)	.108	22/295	12/686	3.02 (1.06‐8.58)	.038
Low‐to‐moderate dose vs control	14/226	12/686	3.09 (0.95‐10.12)	.062	18/226	12/686	3.70 (1.20‐11.34)	.022
High‐dose vs control	3/55	12/686	1.70 (0.23‐12.65)	.605	4/55	12/686	1.70 (0.23‐12.65)	.605
PaO_2_/FiO_2_<300 mm Hg								
Corticosteroids vs control	215/760	62/400	0.67 (0.46‐0.98)	.038	239/760	64/400	0.68 (0.47‐1.00)	.050
Low‐to‐moderate dose vs control	99/436	62/400	0.49 (0.32‐0.77)	.002	112/436	64/400	0.51 (0.33‐0.78)	.002
High‐dose vs control	111/312	62/400	0.88 (0.56‐1.39)	.581	122/312	64/400	0.92 (0.59‐1.44)	.717

CI, confidence interval.

a Shown as Events/Total.

In the subgroup analysis for patients with PaO_2_/FiO_2_ ≥300 mm Hg, corticosteroid therapy resulted in increased 60‐day mortality (aHR 3.02 [95% CI 1.06‐8.58]). In contrast, for patients with PaO_2_/FiO_2_<300 mm Hg, low‐to‐moderate‐dose corticosteroids significantly reduced both 30‐day mortality (aHR 0.49 [95% CI 0.32‐0.77]) and 60‐day mortality (aHR 0.51 [95% CI 0.33‐0.78]), while these patients did not benefit from high‐dose corticosteroids treatment (Table 3). To further overcome the immortal time bias, a time‐dependent covariate Cox regression analysis was conducted with the time duration from disease onset to initiation of corticosteroid treatment being considered, and we obtained similar results (Table S3). In the sensitivity analysis, 722 patients who had potential indications for corticosteroid treatment that may have skewed the results, including asthma, COPD exacerbation, pregnancy/post‐partum, immunosuppressive conditions, or shock, were excluded, but similar results were obtained (Table S4).

### 3.4 Propensity score‐matched case‐control analysis of the effect of corticosteroids on mortality

Propensity scores were calculated using a multivariate logistic regression model that included all the factors listed in Table S5 and Table S6, except for the outcomes, and C‐statistics was 0.839, indicating good model discrimination. Then, 265 propensity score‐matched pairs (Table S5) were generated from the low‐to‐moderate‐dose corticosteroid group (n=662) and control group (n=1086). In the high‐dose corticosteroid group (n=367), 148 patients were matched to 148 cases from the control group (Table S6). The baseline characteristics and treatment regimens except for corticosteroids, including timing of antiviral therapy and timing of hospitalization, were well balanced between the patients in the propensity score‐matched corticosteroid group and that in the control group (Table S5 and Table S6).

**Figure 2:**
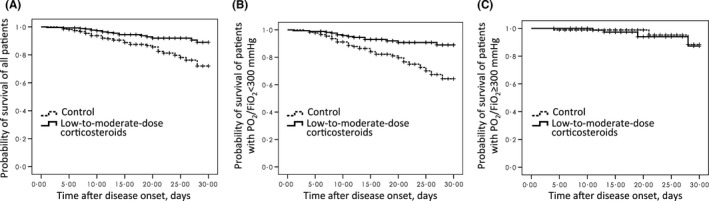
Kaplan‐Meier survival curves for matched patients treated with low‐to‐moderate‐dose corticosteroids or with no corticosteroids (control), censored at 30 d. A, Including all the patients (n=530, log‐rank chi‐squared=10.48, *P*=.001), the 30‐day mortality in the low‐to‐moderate dose corticosteroid group and control group was 6.8% (18/265) and 14.7% (39/265), respectively. B, Including patients with PaO_2_/FiO_2_ <300 mm Hg (n=351, log‐rank chi‐squared=13.24, *P*<.001), the 30‐day mortality in the low‐to‐moderate‐dose corticosteroid group and control group was 8.1% (14/173) and 20.2% (36/178), respectively. C, Including patients with PaO_2_/FiO_2_ ≥300 mm Hg (n=179, log‐rank chi‐squared=0.17, *P*=.68), the 30‐day mortality in the low‐to‐moderate‐dose corticosteroid group and control group was 4.3% (4/92) and 3.4% (3/87), respectively

Kaplan‐Meier survival curves (Figure 2A) for the propensity score‐matched low‐to‐moderate‐dose corticosteroid group and control group showed that 30‐day mortality in the corticosteroid group was significantly lower than that in the control group (log‐rank chi‐squared=10.48, *P*=.001). In subgroup analysis, this difference in mortality between the two groups was even more significant when only patients with PaO_2_/FiO_2_<300 mm Hg on admission were assessed (log‐rank chi‐squared=13.24, *P*<.001; Figure 2B). However, the difference was not significant when only patients with PaO_2_/FiO_2_ ≥300 mm Hg on admission were taken into account (log‐rank chi‐squared=0.17, *P*=.683; Figure 2C). Similar results were observed for survival analysis censored at 60 days (Fig. S1).

**Figure 3:**
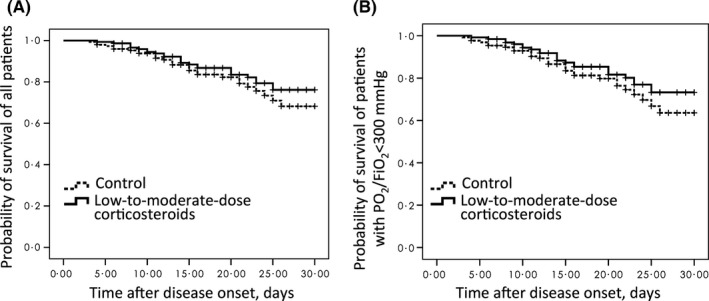
Kaplan‐Meier survival curves for matched patients treated with high‐dose corticosteroids or with no corticosteroids (control), censored at 30 d. A, Including all the patients (n=296, log‐rank chi‐squared=1.06, *P*=.30), the 30‐day mortality in the high‐dose corticosteroid group and control group was 17.6% (26/148) and 19.6% (29/148), respectively. B, Including patients with PaO_2_/FiO_2_ <300 mm Hg (n=256, log‐rank chi‐squared=1.33, *P*=.25), the 30‐day mortality in the high‐dose corticosteroids group and control group was 19.7% (25/127) and 22.5% (29/129), respectively

Neither the 30‐day (Figure 3) nor the 60‐day mortality (Fig. S2) was significantly different between the propensity score‐matched high‐dose corticosteroid group and the control group. Only 20 patients with PaO_2_/FiO_2_ ≥300 mm Hg were given high‐dose corticosteroid therapy, and one death occurred; therefore, subgroup analysis was not feasible for these patients.

### 3.5 Effect of corticosteroid treatment on nosocomial infection

Nosocomial infection occurred 21.5% (227/1055) of patients in the corticosteroid group, which was significantly higher than that in the control group. Compared with the low‐to‐moderate‐dose corticosteroid group, more patients in the high‐dose corticosteroid group experienced nosocomial bacterial infection (16.8% vs 24.8%, *P*=.002). The most common isolated pathogens were *Acinetobacter baumannii*,* Pseudomonas aeruginosa*, and *Staphylococcus aureus* (Table 4).

**Table 4:** Nosocomial infection of all 2141 patients hospitalized with influenza A(H1N1)pdm09 viral pneumonia

	Comparison of patients given different doses of corticosteroids			Comparison of patients given corticosteroids and control		
	High‐dose n=367	Low‐to‐moderate n=662	*P*‐value	Corticosteroids n=1055	Control n=1086	*P*‐value
Bacterial infections	91 (24.8)	111 (16.8)	.002	202 (19.1)	43 (4.1)	<.001
*Acinetobacter baumannii*	25 (6.8)	47 (7.1)	.862	72 (6.8)	13 (1.2)	<.001
*Pseudomonas aeruginosa*	16 (4.4)	13 (2.0)	.026	29 (2.7)	4 (0.4)	<.001
*Staphylococcus aureus*	14 (3.8)	7 (1.1)	.003	21 (2.0)	6 (0.6)	.003
*Stenotrophomonas maltophilia*	8 (2.2)	9 (1.4)	.323	17 (1.6)	1 (0.1)	<.001
*Klebsiella pneumoniae*	8 (2.2)	7 (1.1)	.150	15 (1·4)	2 (0.2)	.001
*Burkholderia cepacia*	3 (0.8)	5 (0.8)	1.000	8 (0.8)	1 (0.1)	.041
*Escherichia coli*	2 (0.5)	5 (0.8)	1.000	7 (0.7)	1 (0.1)	.070
*Enterobacter cloacae*	2 (0.5)	3 (0.5)	1.000	5 (0.5)	2 (0.2)	.426
*Moraxella catarrhalis*	1 (0.3)	1 (0.2)	1.000	2 (0.2)	3 (0.3)	1.000
*Streptococcus pneumoniae*	0 (0.0)	2 (0.3)	.541	2 (0.2)	2 (0.2)	1.000
*Haemophilus influenzae*	2 (0.5)	2 (0.3)	.939	4 (0.4)	0 (0.0)	.126
*Enterobacter aerogenes*	2 (0.5)	2 (0.3)	.939	4 (0.4)	0 (0.0)	.126
Other bacteria	8 (2.2)	8 (1.2)	.228	16 (1.5)	8 (0.7)	.087
Invasive fungal infections	9 (2.5)	16 (2.4)	.972	25 (2.4)	3 (0.3)	<.001

Categorical variables were presented as number (percentage).

Chi‐squared test was used for comparison of patients from different groups.

## 4 DISCUSSION

Using a nationwide database comprising of 2141 hospitalized influenza A(H1N1)pdm09 viral pneumonia patients, among whom 89% were younger than 60 years, we found that severe influenza A(H1N1)pdm09 viral pneumonia patients with PaO_2_/FiO_2_<300 mm Hg might benefit from low‐to‐moderate‐dose corticosteroid. However, for the less severe patients with PaO_2_/FiO_2_ ≥300 mm Hg, corticosteroid therapy might be harmful.

Corticosteroids were not recommended for patients with influenza infection by some guidelines.^20^ A considerable number of observational studies have shown that mortality rates in influenza‐infected patients who received corticosteroid therapy were similar or even higher than that in patients who did not.^17^, ^21^ In this study, univariate analysis also revealed that corticosteroid therapy was associated with increased mortality; however, patients who received corticosteroid therapy had more severe baseline disease (Table S5 and Table S6). Therefore, the higher mortality rate in corticosteroid group might just result from the more severe baseline condition of patients in this group.^17^ One study of influenza A(H1N1)pdm09 virus‐infected patients with ARDS showed that low‐to‐moderate‐dose corticosteroid (1 mg·kg^−1^·d^−1^) was associated with improvement of lung injury and reduced mortality. However, this conclusion was limited by a low number of cases.^9^

To further explore whether corticosteroid therapy at a certain dose range is effective for a certain group of influenza pneumonia patients or not, we performed stratified analysis of patients with different disease severity, based on PaO_2_/FiO_2_ on admission. This stratification is of great importance, because PaO_2_/FiO_2_ is a key factor predicting mortality and may respond to corticosteroid use.^30^, ^37^ In the present study, based on the largest dataset to date, we found that corticosteroids have a tendency to reduce mortality of patients with severe influenza A(H1N1)pdm09 viral pneumonia who have PaO_2_/FiO_2_<300 mm Hg. However, for patients with mild influenza A(H1N1)pdm09 viral pneumonia, with PaO_2_/FiO_2_ ≥300 mm Hg, corticosteroids could not reduce mortality and their use was even associated with a higher 60‐day mortality rate.

Despite being an important factor related to the efficacy of corticosteroids, dosage was mentioned in few studies published to date.^17^, ^21^ In the current study, we further performed stratified analysis based on the doses of corticosteroids and found that only low‐to‐moderate dose of corticosteroids could lower the mortality rate in severe influenza A(H1N1)pdm09 viral pneumonia patients with PaO_2_/FiO_2_<300 mm Hg. The beneficial effect was not noticed in the large‐dose corticosteroid group.

Except for the above factors, difference in study population might be another important element that contributed to the inconsistency between results revealed in this study and that published before.^13^, ^14^ Compared to the study population in previous researches,^17^ patients enrolled in this study were much younger with a median age of just 34.4 years and fewer with underlying diseases. In both Kim and Brun's study, the mean age of patients was more than 10 years older than patients in this study, and more than 80% of them had one or more comorbidities.^13^, ^14^ And further the younger age and fewer underlying disease might lead to fewer secondary infection.^38^, ^39^ Although compared to that in patients who did not receive corticosteroid therapy, the secondary infection rate was higher in the corticosteroid group, especially in the large‐dose corticosteroid group, it was still much lower than that in the study by Kim, which reported that 57% of their total study population complicated secondary bacterial infection.^13^ We further divided the patients into two groups based on their age as well as comorbidities. By multivariate Cox regression analysis, we found that corticosteroid showed no beneficial effect in influenza viral pneumonia patients aged >60 years or with comorbidities, while low‐to‐moderate corticosteroid therapy reduced mortality in severe influenza viral pneumonia patients (PaO_2_/FiO_2_<300 mm Hg) younger than 60 years and without any underlying disease (Table S7). Although the statistical power might not be potent enough to draw a robust conclusion, it gave us some hints that effect of corticosteroid might be influenced by age and underlying diseases of target population.

This study has some limitations. First, the intrinsic defects of retrospective studies were unavoidable, as confounding factors cannot be addressed completely. And the indication of corticosteroids and why the varied doses were used were not well recorded in the database. Although we tried to minimize the selection bias for corticosteroid use among different patients with propensity‐matched analysis, only the known and measured confounders can be adjusted. Second, the effects of corticosteroids on viral shedding could not be assessed due to missing data. Third, in contrast to evaluation of a single dose in an RCT design, the dose in this study ranged from 25 to 150 mg. As body weights were not recorded in detail, doses of corticosteroids could not be calculated based on body weight.

Collectively, although we cannot avoid the possibility that the results might be skewed by the intrinsic limits of retrospective studies, our data should give some hints for future RCT studies. When considering an experimental therapy in influenza pneumonia patients, low‐to‐moderate dose corticosteroid should be tested preferably among the severe viral pneumonia patients with PaO_2_/FiO_2_<300 mm Hg.

## NOTATION OF PUBLICATION

This work has been accepted as a rapid oral poster presentation at the ISIRV‐Options IX for the Control of Influenza Conference on June 24th‐28th, 2016 at the Sheraton Grand Chicago Hotel, Chicago, USA.
