# Supplementary material for: Effect of low‐to‐moderate‐dose corticosteroids on mortality of hospitalized adolescents and adults with influenza A(H1N1)pdm09 viral pneumonia
Source: Influenza Other Respir Viruses. 2017 Jun 9;11(4):345–54. doi: 10.1111/irv.12456 (PMC5485871; doi:10.1111/irv.12456)

**Supplementary Material**

**Effect of low-to-moderate dose corticosteroids on mortality of hospitalized adolescents and adults with influenza A(H1N1)pdm09 viral pneumonia**

Hui Li, MD^1^; Shi-gui Yang, MD^2^; Li Gu, MD^1^; Yao Zhang, MD^3^; Xi-xin Yan, MD^4^; Zong-an Liang, MD^5^; Wei Zhang, MD^6^; Hong-yu Jia, MD^2^; Wei Chen, MD^7^; Meng Liu, MD^1^; Kai-jiang Yu, MD^8^; Chun-xue Xue, MD^1^; Ke Hu, MD^9^; Qi Zou, MD^10^; Lan-juan Li, MD^2^; Bin Cao, MD^11,12,13^; Chen Wang, MD^11,12,13,14^; for the National Influenza A(H1N1)pdm09 Clinical Investigation Group of China

**FIGURE LEGENDS**

**Figure S1. Kaplan−Meier survival curves for matched patients treated with low-to-moderate dose corticosteroids or with no corticosteroids (control), censored at 60 days.**

A. Including all the patients (n = 530, Log-rank Chi-squared = 9.93, p = 0.002), the 60-day mortality in the low-to-moderate dose corticosteroids group and control group were 7.5% (20/265) and 15.1% (40/265), respectively.

B. Including patients with PaO_2_/FiO_2_ < 300 mmHg (n = 351, Log-rank Chi-squared = 13.57, p <0.001), the 60-day mortality in the low-to-moderate dose corticosteroids group and control group were 8.7% (15/173) and 20.8% (37/178), respectively.

C. Including patients with PaO_2_/FiO_2_ ≥ 300 mmHg (n = 179, Log-rank Chi-squared = 0.54, p = 0.46), the 60-day mortality in the low-to-moderate dose corticosteroids group and control group were 5.4% (5/92) and 3.4% (3/87), respectively.

**Figure S2. Kaplan−Meier survival curves for matched patients treated with high-dose corticosteroids or with no corticosteroids, censored at 60 days.**

A. Including all patients (n = 296, Log-rank Chi-squared = 1.04, p = 0.31), the 60-day mortality in the high-dose corticosteroids group and control group were 18.9% (28/148) and 20.3% (30/148), respectively.

B. Including patients with PaO_2_/FiO_2_ < 300 mmHg (n = 256, Log-rank Chi-squared = 1.29, p = 0.26), the 60-day mortality in the high-dose corticosteroids group and control group were 21.3% (27/127) and 23.3% (30/129), respectively.

**Table S1. Distribution of patients by province in mainland China**

| **Patients' location** | **Number of hospitals**  **n (%)** | **Number of patients**  **n (%)** |
| --- | --- | --- |
| Hebei Province | 48 (11.8) | 354 (16.5) |
| Beijing | 36 (8.8) | 247 (11.5) |
| Zhejiang Province | 54 (13.3) | 225 (10.5) |
| Liaoning Province | 26 (6·4) | 210 (9.8) |
| Shandong Province | 35 (8.6) | 170 (7.9) |
| Sichuan Province | 35 (8.6) | 168 (7.8) |
| Gansu Province | 24 (5.9) | 107 (5.0) |
| Fujian Province | 21 (5.2) | 83 (3.9) |
| Hunan Province | 21 (5.2) | 75 (3.5) |
| Jilin Province | 11 (2.7) | 69 (3.2) |
| Shanghai | 9 (2.2) | 67 (3.1) |
| Jiangxi Province | 18 (4.4) | 56 (2.6) |
| Heilongjiang Province | 12 (2.9) | 46 (2.1) |
| Anhui Province | 3 (0.7) | 43 (2.0) |
| Hubei Province | 2 (0.5) | 42 (2.0) |
| Guangxi Zhuang Autonomous Region | 13 (3.2) | 34 (1.6) |
| NingXia Hui Autonomous Region | 2 (0.5) | 33 (1.5) |
| Inner Mongolia Autonomous Region | 13 (3.2) | 30 (1.4) |
| Xinjiang Uygur Autonomous Region | 6 (1.5) | 19 (0.9) |
| Guizhou Province | 7 (1.7) | 14 (0.7) |
| Chongqing | 1 (0.2) | 12 (0.6) |
| Guangdong Province | 4 (1.0) | 12 (0.6) |
| Shanxi Province（陕西） | 2 (0.5) | 11 (0.5) |
| The Tibet Autonomous Region | 1 (0.2) | 8 (0.4) |
| Yunnan Province | 1 (0.2) | 4 (0.2) |
| Tianjin | 1 (0.2) | 1 (0.0) |
| Shanxi Province(山西) | 1 (0.2) | 1(0.0) |
| Total | 407 | 2141 |

Categorical variables are presented as number (percentage).

**Table S2. Characteristics of 2141 patients hospitalized with influenza A(H1N1)pdm09 viral pneumonia stratified according to the patients' final outcome**

|  | **All patients**  **(n = 2141)** | **Deaths**  **(n = 340)** | **Survivors**  **(n = 1801)** | **P-value*** |
| --- | --- | --- | --- | --- |
| Hospital category |  |  |  |  |
| Secondary hospital | 254 (11.9) | 29 (8.5) | 225 (12.5) | 0.038 |
| Tertiary hospital | 1887 (88.1) | 311 (91.5) | 1576 (87.5) |  |
| Age, year | 34.4 (24.1−51.1) | 35.4 (24.4−53.5) | 34.3 (24−50.8) | 0.220 |
| 14−20 | 271 (12.7) | 38 (11.2) | 233 (12.9) | 0.203 |
| 21−40 | 1049 (49) | 169 (49.7) | 880 (48.9) |  |
| 41−60 | 579 (27) | 83 (24.4) | 496 (27.5) |  |
| 61−80 | 218 (10.2) | 46 (13.5) | 172 (9.6) |  |
| > 80 | 24 (1.1) | 4 (1.2) | 20 (1.1) |  |
| Female | 1046 (48.9) | 166 (48.8) | 880 (48.9) | 0.990 |
| Underlying diseases |  |  |  |  |
| Hypertension | 326 (15.2) | 52 (15.3) | 274 (15.2) | 0.970 |
| Diabetes | 168 (7.8) | 28 (8.2) | 140 (7.8) | 0.771 |
| Cardiovascular disease^†^ | 131 (6.1) | 27 (7.9) | 104 (5.8) | 0.126 |
| COPD | 106 (5) | 20 (5.9) | 86 (4.8) | 0.388 |
| Asthma | 38 (1.8) | 3 (0.9) | 35 (1.9) | 0.174 |
| Chronic renal disease | 74 (3.5) | 22 (6.5) | 52 (2.9) | 0.001 |
| Malignancy^‡^ | 54 (2.5) | 17 (5) | 37 (2.1) | 0.001 |
| Immunosuppressive conditions^§^ | 49 (2.3) | 12 (3.5) | 37 (2.1) | 0.096 |
| Pregnancy or postpartum | 452 (21.1) | 92 (27.1) | 360 (20) | 0.003 |
| Main laboratory findings on admission |  |  |  |  |
| Leukocytosis | 345/2092 (16.5) | 85/334 (25.4) | 260/1758 (14.8) | <0.001 |
| Leukocytopenia | 527/2092 (25.2) | 83/334 (24.9) | 444/1758 (25.3) | 0.876 |
| Lymphocytopenia | 852/2009 (42.4) | 196/317 (61.8) | 656/1692 (38.8) | <0.001 |
| Hemoglobin, g/L | 128 (108−144) | 118.5 (96.8−140) | 129 (110−145) | <0.001 |
| Platelets, 10^9^/L | 152 (113−200) | 136.5 (97.5−180) | 154 (118−205) | <0.001 |
| Creatinine, µmol/L | 70 (54.5−88) | 76.5 (60.9−107.9) | 69 (54−86) | <0.001 |
| PaO_2_/FiO_2_, mmHg | 219.5 (127.9−326.4) | 112.9 (73.8−198.7) | 248.4 (157.1-353.9) | <0.001 |
| Alanine aminotransferase, U/L | 29 (17−51) | 36 (21−71.3) | 28 (17−48.6) | <0.001 |
| Lactate dehydrogenase, U/L | 316 (213−542) | 611 (325.5−963.5) | 295 (203.1−467) | <0.001 |
| Shock | 131/2132 (6.1) | 38/337 (11.3) | 93/1795 (5.2) | <0.001 |
| Invasive mechanical ventilation | 416 (19.4) | 239 (70.3) | 177 (9.8) | <0.001 |
| NAI treatment | 2047 (95.6) | 317 (93.2) | 1730 (96.1) | 0.020 |
| Antibiotics | 2092 (97.7) | 336 (98.8) | 1756 (97.5) | 0.135 |
| Interval between symptom onset and hospitalization, days | 5 (3−7) | 6 (4−7) | 5 (3−7) | 0.018 |
| Corticosteroids | 1055 (49.3) | 262 (77.1) | 793 (44.0) | <0.001 |
| Low-to-moderate dose | 662 (29.3) | 130 (38.2) | 532 (29.5) | 0.001 |
| High-dose | 367 (17.1) | 127 (37.4) | 240 (13.3) | <0.001 |

Definition of abbreviations: COPD = chronic obstructive pulmonary disease. NAI = Neuraminidase inhibitors.

Continuous variables are summarized as median (interquartile range), and categorical variables are presented as number (percentage).

*Mann−Whitney U tests for continuous variables and the Chi-square tests for categorical variables.

^†^Cardiovascular disease: including congestive heart disease, coronary atherosclerotic heart disease, and valvular heart disease.

^‡^Malignancy: cancer or hematologic malignancy.

^§^Immunosuppressive condition: chemotherapy or radiotherapy within 1 month before the onset of illness, or glucocorticoid therapy (equivalent of 30 mg of prednisone per day for 15 continuous days before the onset of illness.

**Table S3. Effect of corticosteroids on mortality in 2141 patients hospitalized with influenza A(H1N1)pdm09 viral pneumonia by time-dependent covariate Cox regression analysis**

|  | **30-Day Mortality** | |  | **60-Day Mortality** | |
| --- | --- | --- | --- | --- | --- |
|  | **Adjust HR (95%CI)** | **P-value** |  | **Adjust HR (95%CI)** | **P-value** |
| **All patients** |  |  |  |  |  |
| Corticosteroids vs. control | 0.573 (0.298−1.103) | 0.10 |  | 0.651 (0.310−1.368) | 0.26 |
| Low-to-moderate dose vs. control | 0.386 (0.183−0.812) | 0.012 |  | 0.445(0.189−1.049) | 0.06 |
| High-dose vs. control | 0.872 (0.400−1.900) | 0.73 |  | 1.021 (0.439−2.377) | 0.96 |
| **PaO_2_/FiO_2_≥300 mmHg** |  |  |  |  |  |
| Corticosteroids vs. control | 1.596 (0.264−9.652) | 0.61 |  | 3.523 (0.382−32.459) | 0.27 |
| Low-to-moderate dose vs. control | 1.701 (0.250−11.577) | 0.59 |  | 3.943 (0.392−39.705) | 0.24 |
| High-dose vs. control | 10.027 (0.210−477.8) | 0.24 |  | 10.027 (0.210−477.8) | 0.24 |
| **PaO_2_/FiO_2_<300 mmHg** |  |  |  |  |  |
| Corticosteroids vs. control | 0.459 (0.226−0.935) | 0.032 |  | 0.501 (0.227−1.104) | 0.09 |
| Low-to-moderate dose vs. control | 0.310 (0.137−0.705) | 0.005 |  | 0.338 (0.133−0.862) | 0.023 |
| High-dose vs. control | 0.751 (0.335−1.686) | 0.49 |  | 0.841 (0.348−2.032) | 0.70 |

**Table S4. Effect of corticosteroids on mortality in 1419 patients* hospitalized with influenza A(H1N1)pdm09 viral pneumonia by multivariate Cox regression analysis**

|  | **30-Day Mortality** | |  | **60-Day Mortality** | |
| --- | --- | --- | --- | --- | --- |
|  | **Adjust HR (95%CI)** | **P-value** |  | **Adjust HR (95%CI)** | **P-value** |
| **All patients** |  |  |  |  |  |
| Corticosteroids vs. control | 0.873 (0.573−1.331) | 0.53 |  | 0.949 (0.627−1.438) | 0.81 |
| Low-to-moderate dose vs. control | 0.729 (0.453−1.172) | 0.19 |  | 0.790 (0.495−1.260) | 0.32 |
| High-dose vs. control | 0.942 (0.551−1.613) | 0.83 |  | 1.025 (0.607−1.732) | 0.93 |
| **PaO_2_/FiO_2_≥300 mmHg** |  |  |  |  |  |
| Corticosteroids vs. control | 3.053 (0.721−12.922) | 0.13 |  | 4.290 (1.090−16.881) | 0.037 |
| Low-to-moderate dose vs. control | 3.613 (0.802−16.278) | 0.09 |  | 5.635 (1.309−24.261) | 0.020 |
| High-dose vs. control | 3.382 (0.294−38.874) | 0.33 |  | 3.382 (0.294−38.874) | 0.33 |
| **PaO_2_/FiO_2_<300 mmHg** |  |  |  |  |  |
| Corticosteroids vs. control | 0.656 (0.412−1.045) | 0.08 |  | 0.661 (0.415−1.050) | 0.08 |
| Low-to-moderate dose vs. control | 0.508 (0.296−0.871) | 0.014 |  | 0.511 (0.299−0.875) | 0.014 |
| High-dose vs. control | 0.781 (0.440−1.384) | 0.40 |  | 0.805 (0.456−1.418) | 0.45 |

*Patients with asthma, chronic obstructive pulmonary disease, pregnancy or postpartum, immunosuppressive conditions, or shock were excluded.

**Table S5. Baseline characteristics of influenza A(H1N1)pdm09 viral pneumonia patients who received low-to-moderate dose corticosteroids or no corticosteroids therapy in unmatched and propensity score-matched groups**

|  | **Unmatched groups** | | |  | **Matched groups** | | |
| --- | --- | --- | --- | --- | --- | --- | --- |
|  | **Low-to-moderate dose corticosteroids**  **n = 662** | **Control**  **n = 1086** | **P-value*** |  | **Low-to-moderate dose corticosteroids**  **n = 265** | **Control**  **n = 265** | **P-value^†^** |
| Hospital category |  |  |  |  |  |  |  |
| Secondary hospital | 73 (11.0) | 137 (12.6) | 0.32 |  | 31 (11.7) | 32 (12.1) | 0.89 |
| Tertiary hospital | 589 (89.0) | 949 (87.4) |  |  | 234 (88.3) | 233 (87.9) |  |
| Age, years | 35 (25.0−52.1) | 35 (23.8−52.4) | 0.33 |  | 35.9 (26.6−55.0) | 38.8 (25.1−53.0) | 0.94 |
| 14−20 | 67 (10.1) | 162 (14.9) | 0.012 |  | 19 (7.2) | 19 (7.2) | 0.90 |
| 21−40 | 342 (51.7) | 481 (44.3) |  |  | 134 (50.6) | 125 (47.1) |  |
| 41−60 | 171 (25.8) | 306 (28.2) |  |  | 75 (28.3) | 85 (32.1) |  |
| 61-80 | 74 (11.2) | 124 (11.4) |  |  | 33 (12.5) | 33 (12.5) |  |
| >−80 | 8 (1.2) | 13 (1.2) |  |  | 4 (1.5) | 3 (1.1) |  |
| Female | 324 (48.9) | 521 (48.0) | 0.69 |  | 126 (47.5) | 137 (51.7) | 0.34 |
| Underlying diseases |  |  |  |  |  |  |  |
| Hypertension | 104 (15.7) | 168 (15.5) | 0.89 |  | 40 (15.1) | 50 (18.9) | 0.20 |
| Diabetes | 48 (7.3) | 90 (8.3) | 0.44 |  | 20 (7.5) | 25 (9.4) | 0.44 |
| Cardiovascular disease^‡^ | 42 (6.3) | 75 (6.9) | 0.65 |  | 17 (6.4) | 27 (10.2) | 0.12 |
| COPD | 45 (6.8) | 47 (4.3) | 0.025 |  | 20 (7.5) | 23 (8.7) | 0.75 |
| Asthma | 16 (2.4) | 16 (1.5) | 0.15 |  | 9 (3.4) | 6 (2.3) | 0.58 |
| Chronic renal disease | 26 (3.9) | 38 (3.5) | 0.64 |  | 8 (3.0) | 11 (4.2) | 0.63 |
| Malignancy^§^ | 14 (2.1) | 26 (2.4) | 0.71 |  | 5 (1.9) | 2 (0.8) | 0.45 |
| Immunosuppressive conditions^\|\|^ | 24 (3.6) | 15 (1.4) | 0.002 |  | 9 (3.4) | 6 (2.3) | 0.61 |
| Pregnancy or postpartum | 154 (23.3) | 182 (16.8) | <0.001 |  | 51 (19.2) | 55 (20.8) | 0.67 |
| Main laboratory findings on admission |  |  |  |  |  |  |  |
| Leukocytes, 10^9^/L | 6 (4.0−8.7) | 5.7 (4.0−8.3) | 0.41 |  | 5.9 (4.1−8.5) | 5.8 (3.9−8.7) | 0.88 |
| Lymphocytes, 10^9^/L | 0.8 (0.5−1.1) | 1.1 (0.7−1.5) | <0.001 |  | 0.9 (0.6−1.2) | 0.9 (0.6−1.2) | 0.79 |
| Hemoglobin, g/L | 127 (105.0−144.0) | 129 (109.0−143.0) | 0.30 |  | 129.0 (109.0−145.0) | 125 (105.0−141.0) | 0.07 |
| Platelets, 10^9^/L | 142 (104.3−194.8) | 163 (125.0−209.0) | <0.001 |  | 151.0 (113.0−210.0) | 166 (126.0−209.0) | 0.07 |
| Creatinine, µmol/L | 69 (54.0−90.0) | 71 (55.3−86.9) | 0.64 |  | 67.4 (54.0−87.0) | 71.1 (55.4−88.0) | 0.30 |
| PaO_2_/FiO_2_, mmHg | 206.5 (123.7−298.6) | 286·2 (191.7−388.2) | <0.001 |  | 251.5 (163.8−338.1) | 244 (162.1−333.3) | 0.31 |
| Alanine aminotransferase, U/L | 33 (20.4−59.8) | 24 (15.0−43.0) | <0.001 |  | 29 (18.5−55.0) | 29 (17.0−51.0) | 0.50 |
| Lactate dehydrogenase, U/L | 403.5 (245.0−656.8) | 257 (184.0−372.9) | <0.001 |  | 353 (228.9−574.0) | 322 (230.1−489.0) | 0.09 |
| Shock | 41 (6.2) | 47 (4.4) | 0.09 |  | 16 (6.0) | 19 (7.2) | 0.72 |
| Invasive mechanical ventilation | 201 (30.4) | 49 (4.5) | <0.001 |  | 37 (14.0) | 36 (13.6) | 1.00 |
| NAI treatment |  |  |  |  |  |  |  |
| none | 22 (3.3) | 64 (5·9) | <0.001 |  | 2 (0.8) | 9 (3.4) | 0.44 |
| > 5 days | 362 (54.8) | 449 (41.3) |  |  | 134 (50.6) | 137 (51.7) |  |
| 3−5 days | 209 (31.6) | 424 (39.0) |  |  | 99 (37.4) | 87 (32.8) |  |
| 0−2 days | 68 (10.3) | 149 (13.7) |  |  | 30 (11.3) | 32 (12.1) |  |
| Time from symptom onset to antiviral therapy, days | 6 (4−8) | 5 (3−7) | <0.001 |  | 6 (4−7) | 6 (4−8) | 0.39 |
| Antibiotic treatment | 653 (98.6) | 1055 (97.1) | 0.043 |  | 263 (99.2) | 261 (98.5) | 0.68 |
| Time from symptom onset to hospitalization, days | 5 (3−7) | 5 (3−7) | 0.004 |  | 5 (4−7) | 5 (3−7) | 0.47 |
| 30-day mortality | 113 (17.1) | 74 (6.8) | <0.001 |  | 18 (6.8) | 39 (14.7) | 0.002 |
| 60-day mortality | 130 (19.6) | 76 (7.0) | <0.001 |  | 20 (7.5) | 40 (15.1) | 0.004 |

Continuous variables are summarized as median (interquartile ranges), and categorical variables are presented as number (percentage).

Definition of abbreviations: COPD = chronic obstructive pulmonary disease. NAI = Neuraminidase inhibitors.

^*^Mann−Whitney U tests for continuous variables, and the Chi-square test for categorical variables.

^†^Wilcoxon signed rank test for continuous variables, and McNemar's test for categorical variables.

^‡^Cardiovascular disease: including congestive heart disease, coronary atherosclerotic heart disease, valvular heart disease.

^§^Malignancy: cancer or hematologic malignancy.

^||^Immunosuppressive conditions: chemotherapy or radiotherapy within 1 month before the onset of illness, or glucocorticoid therapy (equivalent of 30 mg of prednisone per day for 15 continuous days before the onset of illness).

**Table S6. Baseline characteristics of influenza A(H1N1)pdm09 viral pneumonia patients who received high-dose corticosteroids or no corticosteroids therapy in unmatched and propensity score-matched groups**

|  | **Unmatched groups** | | |  | **Matched groups** | | |
| --- | --- | --- | --- | --- | --- | --- | --- |
|  | **High-dose Corticosteroids**  **n = 367** | **Control**  **n = 1086** | **P-value*** |  | **High-dose Corticosteroids**  **n = 148** | **Control**  **n = 148** | **P-value^†^** |
| Hospital category |  |  |  |  |  |  |  |
| Secondary hospital | 40 (10.9) | 137 (12.6) | 0.38 |  | 15 (10.1) | 11 (7.4) | 0.54 |
| Tertiary hospital | 327 (89.1) | 949 (87.4) |  |  | 133 (89.9) | 137 (92.6) |  |
| Age, year | 31.6 (23.9−43.9) | 35.0 (23.8−52.4) | 0.033 |  | 35 (25.3−47.5) | 33.2 (24.5−46.3) | 0.37 |
| 14−20 | 40 (10.9) | 162 (14.9) | <0.001 |  | 11 (7.4) | 17 (11.5) | 0.45 |
| 21−40 | 213 (58.0) | 481 (44.3) |  |  | 82 (55.4) | 85 (57.4) |  |
| 41−60 | 93 (25.3) | 306 (28.2) |  |  | 44 (29.7) | 33 (22.3) |  |
| 61−80 | 19 (5.2) | 124 (11.4) |  |  | 9 (6.1) | 12 (8.1) |  |
| > 80 | 2 (0.5) | 13 (1.2) |  |  | 2 (1.4) | 1 (0.7) |  |
| Female | 190 (51.8) | 521 (48.0) | 0.21 |  | 75 (50.7) | 75 (50.7) | 1.00 |
| Underlying diseases |  |  |  |  |  |  |  |
| Hypertension | 51 (13.9) | 168 (15.5) | 0.47 |  | 28 (8.9) | 22 (14.9) | 0.36 |
| Diabetes | 27 (7.4) | 90 (8.3) | 0.57 |  | 13 (8.8) | 8 (5.4) | 0.38 |
| Cardiovascular disease^‡^ | 13 (3.5) | 75 (6.9) | 0.020 |  | 6 (4.1) | 11 (7.4) | 0.30 |
| COPD | 12 (3.3) | 47 (4.3) | 0.37 |  | 6 (4.1) | 7 (4.7) | 1.00 |
| Asthma | 3 (0.8) | 16 (1.5) | 0.49 |  | 1 (0.7) | 2 (1.4) | 1.00 |
| Chronic renal disease | 10 (2.7) | 38 (3.5) | 0.47 |  | 4 (2.7) | 2 (1.4) | 0.68 |
| Malignancy^§^ | 11 (3.0) | 26 (2.4) | 0.53 |  | 4 (2.7) | 3 (2.0) | 1.00 |
| Immunosuppressive conditions^\|\|^ | 7 (1.9) | 15 (1.4) | 0.47 |  | 2 (1.4) | 2 (1.4) | 0.62 |
| Pregnancy or postpartum | 113 (30.8) | 182 (16.8) | <0.001 |  | 43 (29.1) | 32 (21.6) | 0.14 |
| Main lab-findings on admission |  |  |  |  |  |  |  |
| Leukocytes, 10^9^/L | 5.4 (3.8−8.3) | 5.7 (4.0−8.3) | 0.25 |  | 5.6 (3.9−8.3) | 5.2 (3.5−8.5) | 0.59 |
| Lymphocytes, 10^9^/L | 0.7 (0.4−1.0) | 1.1 (0.7−1.5) | <0.001 |  | 0.7 (0.4−1.1) | 0.8 (0.5−1.2) | 0.053 |
| Hemoglobin, g/L | 127 (105−145) | 129 (109−143) | 0.63 |  | 128 (107−145) | 128 (102.5−141.5) | 0.74 |
| Platelets, 10^9^/L | 140 (104.5−180.5) | 163 (125−209) | <0.001 |  | 152 (122−192.5) | 151 (112−194.5) | 0.87 |
| Creatinine, µmol/L | 69 (54−92.6) | 70.9 (55.3−86.8) | 0.83 |  | 69 (54.9−88.7) | 70.8 (54.8-88.4) | 0.57 |
| PaO_2_/FiO_2_, mmHg | 124.5 (79.1−209.6) | 286.2 (191.7−388.2) | <0.001 |  | 181.1 (116.3−262) | 183·6 (102.1−246.9) | 0.67 |
| Alanine aminotransferase, U/L | 39 (24.5−69) | 24 (15−43) | <0.001 |  | 33.5 (23.2−56.5) | 30.5 (18.7−63.6) | 0.51 |
| Lactate dehydrogenase, U/L | 516 (338−789) | 257 (184−372) | <0.001 |  | 435.8 (283.5-660) | 413 (261.2−575.5) | 0.09 |
| Shock | 42 (11.5) | 47 (4.4) | <0.001 |  | 9 (6.1) | 14 (9.5) | 0.38 |
| Invasive mechanical ventilation | 163 (44.4) | 49 (4.5) | <0.001 |  | 28 (18.9) | 28 (18.9) | 0.85 |
| NAI treatment |  |  |  |  |  |  |  |
| None | 6 (1.6) | 64 (5.9) | <0·0001 |  | 1 (0.7) | 7 (4.7) | 0.69 |
| > 5 days | 205 (56.0) | 449 (41.3) |  |  | 85 (57.4) | 80 (54.1) |  |
| 3−5 days | 137 (37.4) | 424 (39.0) |  |  | 50 (33.8) | 47 (31.8) |  |
| 0−2 days | 18 (4.9) | 149 (13−7) |  |  | 12 (8.1) | 14 (9.5) |  |
| Time from symptom onset to antiviral therapy, days | 6 (4−7) | 5 (3−7) | <0.001 |  | 6 (4-7) | 6 (4−8) | 0.82 |
| Antibiotic treatment | 360 (98.1) | 1055 (97.1) | 0.33 |  | 141 (95.3) | 145 (98.0) | 0.29 |
| Time from symptom onset to hospitalization, days | 5 (4−7) | 5 (3-7) | <0.001 |  | 6 (4−7) | 6 (4−7) | 0.94 |
| 30-day mortality | 114 (31.1) | 74 (6.8) | <0.001 |  | 26 (17.6) | 29 (19.6) | 0.87 |
| 60-day mortality | 126 (34.3) | 76 (7.0) | <0.001 |  | 28 (18.9) | 30 (20.3) | 0.73 |

Continuous variables are summarized as median (interquartile ranges), and categorical variables are presented as number (percentage).

Definition of abbreviations: COPD = chronic obstructive pulmonary disease. NAI = Neuraminidase inhibitors.

^*^Mann−Whitney U tests for continuous variables, and the Chi-square test for categorical variables.

^†^Wilcoxon signed rank test for continuous variables, and McNemar's test for categorical variables.

^‡^Cardiovascular disease: including congestive heart disease, coronary atherosclerotic heart disease, valvular heart disease.

^§^Malignancy: cancer or hematologic malignancy.

^||^Immunosuppressive conditions: chemotherapy or radiotherapy within 1 month before the onset of illness, or glucocorticoid therapy (equivalent of 30 mg of prednisone per day for 15 continuous days before the onset of illness).

**Table S7. Effect of corticosteroids on mortality in patients hospitalized with influenza A(H1N1)pdm09 viral pneumonia by multivariate Cox regression analysis (30-day mortality)**

|  | **Patients 60 years old or younger and without any comorbidities** | |  | **Patients older than60 years or with comorbidities** | |
| --- | --- | --- | --- | --- | --- |
|  | **Adjust HR (95%CI)** | **P-value** |  | **Adjust HR (95%CI)** | **P-value** |
| **All patients** |  |  |  |  |  |
| Corticosteroids vs. control | 0.800 (0.437−1.467) | 0.47 |  | 1.062 (0.683−1.652) | 0.79 |
| Low-to-moderate dose vs. control | 0.642 (0.333−1.242) | 0.19 |  | 0.864 (0.529−1.409) | 0.56 |
| High-dose vs. control | 0.822 (0.401−1.686) | 0.59 |  | 1.563 (0.893−2.734) | 0.12 |
| **PaO_2_/FiO_2_<300 mmHg** |  |  |  |  |  |
| Corticosteroids vs. control | 0.649 (0.354−1.188) | 0.16 |  | 1.018 (0.629−1.649) | 0.94 |
| Low-to-moderate dose vs. control | 0.505 (0.266−0.959) | 0.037 |  | 0.763 (0.445−1.310) | 0.76 |
| High-dose vs. control | 0.745 (0.366−1.517) | 0.42 |  | 1.651 (0.914−2.958) | 0.10 |

Patients’ number in PaO_2_/FiO_2_≥300 mmHg group was not enough to perform multivariate Cox regression analysis.

**Figure S1**

**
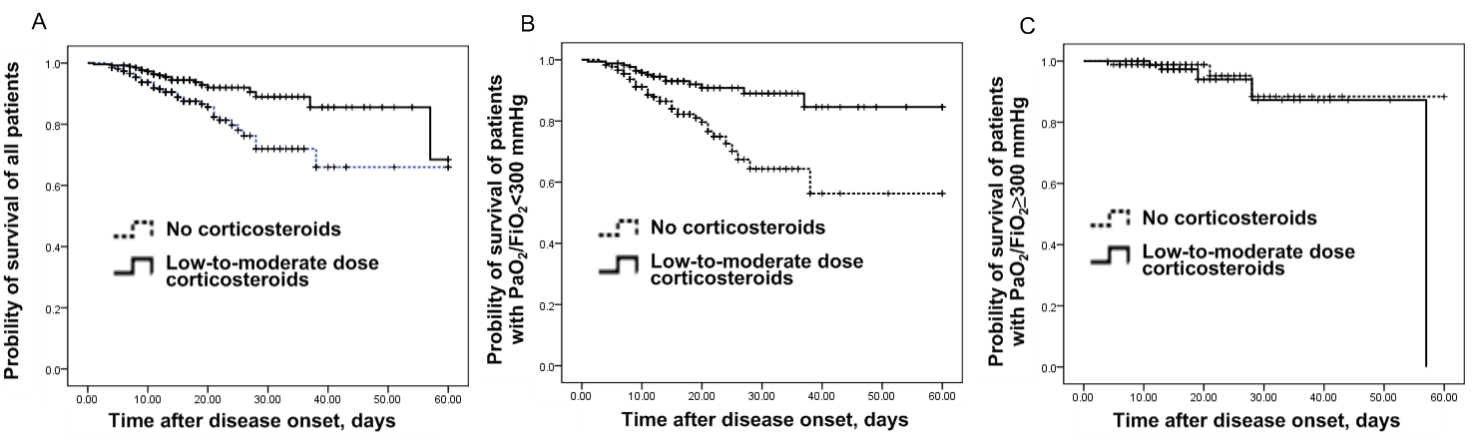
**

**Figure S2**


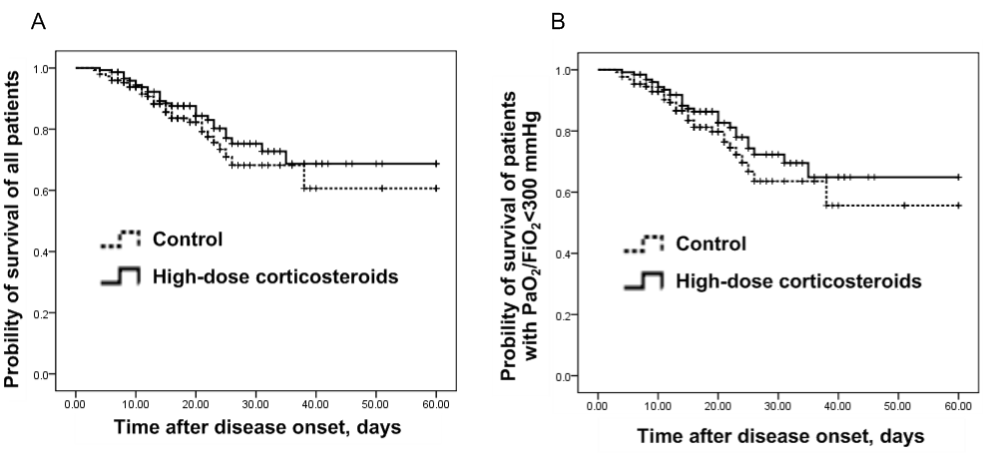

Supplement: Supplementary file 1 [file IRV-11-345-s001.docx]
